# Acetogenesis: An annotated selection of World Wide Web sites relevant to the topics in environmental microbiology

**DOI:** 10.1111/1758-2229.13199

**Published:** 2023-09-01

**Authors:** Lawrence P. Wackett

**Affiliations:** ^1^ McKnight Professor, Department of Biochemistry, Molecular Biology & Biophysics BioTechnology Institute, University of Minnesota St. Paul MN 55108 USA

## Acetogenesis – An overview

### https://www.sciencedirect.com/topics/engineering/acetogenesis

This compendium on acetogenesis focuses on biogas and anaerobic biomass conversions by acetogenic processes.

## Acetogen – An overview

### https://www.sciencedirect.com/topics/agricultural-and-biological-sciences/acetogen

This collection of abstracts, tables and figures focuses on acetogenic organisms. It offers a large table of acetogenic organisms, a phylogenetic tree and a list of acetogens for which genomes are available.

## Energy barriers in acetogenesis

### https://www.frontiersin.org/articles/10.3389/fbioe.2020.621166/full

This review article examines what's known about how acetogens generate energy and it posits ways to overcome energy barriers.

## Energy conservation in acetogens

### https://www.nature.com/articles/nrmicro3365

While this review dates from nine years ago, it still offers a useful and comprehensive treatment of energy conservation by acetogenic bacteria.

## Acetogenesis by methanotrophic archaea

### https://www.nature.com/articles/s41467-020-17860-8

This paper describes isotope labelling and other experiments to propose a model of carbon cycling in marine cold seeps in which acetate is produced during anaerobic oxidation of methane.

## Acetogenesis by an endosymbiont

### https://www.pnas.org/doi/10.1073/pnas.1423979112

This study examined acetogenesis by a symbiont of cellulolytic protists residing in the gut of termites.

## Synthetic biology with acetogens

### https://www.mdpi.com/1422-0067/21/20/7639

Synthesis gas, largely single carbon gases derived from fossil fuels, is discussed here as a substrate for acetogenic bacteria and is used in engineered systems to produce value‐added chemicals.

## Translational regulation of acetogenesis

### https://journals.asm.org/doi/10.1128/msystems.00696-21

This study examined transcriptional and translational aspects of gene expression in *Acetobacterium woodii* under both autotrophic and heterotrophic growth conditions.

## Acetogenesis pathways and proteins

### https://www.wikipathways.org/pathways/WP5019.html

This WikiPathways page shows metabolite maps and has a linked table of metabolites and relevant proteins.

## Crystal structure of acetyl‐CoA synthase, 1MJG

### https://www.rcsb.org/structure/1MJG

This page of the Protein DataBank links to the crystal structure of the bifunctional carbon monoxide dehydrogenase, also known as acetyl‐CoA synthase, from *Moorella thermoacetica*.

## Monomeric acetyl‐CoA synthase

### https://www.rcsb.org/structure/7NYS

This page of the Protein DataBank links to the crystal structure of the monomeric acetyl‐CoA synthase, with carbon monoxide bound to the protein nickel atom. The enzyme is originally derived from *Carboxythermus hydrogenoformans* Z‐2901.

## Homo‐acetogens

### https://www.ncbi.nlm.nih.gov/pmc/articles/PMC8875654/

This paper focuses on homo‐acetogens, which are comprise of many different genera of bacteria, including *Acetobacterium, Clostridium, Morella, Eubacterium, Sporomusa* and *Ruminococcus*.

## Reprogramming acetogenesis

### https://www.nature.com/articles/s41396-023-01411-2

This study describes a genetic knockout of the last step in methyl group oxidation in *Acetobacterium woodii*, leading to the production of formate. This is stated to be the first report of reprogramming of an acetogen into what they denoate as a “formatogen.”

## Carbon fixation map: KEGG

### https://www.genome.jp/pathway/map00720

This is a pathway map which shows the positions of acetogenesis within a cluster of carbon dioxide fixation pathways depicted from a range of organisms.

## Acetogens using light‐driven electrons

### https://www.pnas.org/doi/10.1073/pnas.2020552118

This study showed that an acetogenic bacterium, *Clostridium authoethanogenum*, could use accept electrons from cadmium sulfide nanoparticles activated by light exposure.

## Webinar on acetogenic organisms

### https://www.youtube.com/watch?v=yZhqfvGHPGM

This webinar, sponsored by the Federation of European Microbiology Societies (FEMS) explores various aspects of acetogenic bacteria.

## Why sequence new acetogens?

### https://jgi.doe.gov/why-sequence-novel-acetogenic-bacterial-isolates-from-dechlorinating-microbial-mixed-cultures/

This is a Department of Energy project to DNA sequence acetogenic bacterial isolates from dechlorinating mixed cultures.

